# Towards Reliable Parameter Extraction in MEMS Final Module Testing Using Bayesian Inference

**DOI:** 10.3390/s22145408

**Published:** 2022-07-20

**Authors:** Monika E. Heringhaus, Yi Zhang, André Zimmermann, Lars Mikelsons

**Affiliations:** 1 Robert Bosch GmbH, 72762 Reutlingen, Germany; 2 Institute for Micro Integration (IFM), University of Stuttgart, 70569 Stuttgart, Germany; zimmermann@ifm.uni-stuttgart.de; 3 Chair of Mechatronics, Augsburg University, 86159 Augsburg, Germany; lars.mikelsons@uni-a.de; 4 Hahn-Schickard, 70569 Stuttgart, Germany

**Keywords:** MEMS testing, parameter extraction, uncertainty quantification, Bayesian inference, BayesFlow

## Abstract

In micro-electro-mechanical systems (MEMS) testing high overall precision and reliability are essential. Due to the additional requirement of runtime efficiency, machine learning methods have been investigated in recent years. However, these methods are often associated with inherent challenges concerning uncertainty quantification and guarantees of reliability. The goal of this paper is therefore to present a new machine learning approach in MEMS testing based on Bayesian inference to determine whether the estimation is trustworthy. The overall predictive performance as well as the uncertainty quantification are evaluated with four methods: Bayesian neural network, mixture density network, probabilistic Bayesian neural network and BayesFlow. They are investigated under the variation in training set size, different additive noise levels, and an out-of-distribution condition, namely the variation in the damping factor of the MEMS device. Furthermore, epistemic and aleatoric uncertainties are evaluated and discussed to encourage thorough inspection of models before deployment striving for reliable and efficient parameter estimation during final module testing of MEMS devices. BayesFlow consistently outperformed the other methods in terms of the predictive performance. As the probabilistic Bayesian neural network enables the distinction between epistemic and aleatoric uncertainty, their share of the total uncertainty has been intensively studied.

## 1. Introduction

Testing of MEMS is a challenging task due to the complexity of the systems, especially with the progressing miniaturization of the devices. On top of that, the test process is heavily time critical for economic reasons. Nevertheless, ensuring the robustness of parameter extraction methods is crucial, because calibration and fabrication process control require reliable determination of the system as well as process-related properties, which are often not directly measurable. For parameter identification from dynamic tests, as they are carried out during the final module testing of capacitive MEMS accelerometers, approaches based on numerical solutions are generally most desirable in terms of interpretability of the test results. However, due to nonlinear couplings and inhomogeneities in the system differential equations, the computation time of these approaches exceeds targeted limits especially for overdamped systems. Therefore, data-driven approaches have been suggested for the parameter extraction [1,2,3], which results in a considerable reduction of the inference time. Parameter identification approaches applying machine learning (ML) methods, however, lack the reliability and interpretability of numerical methods. Thus, cautious evaluation of the models is necessary before deployment. Furthermore, the unknown behavior of data-driven approaches beyond their generalization regions usually makes a validation step necessary, for example in the form of calculating an ordinary differential Equation (ODE) solution based on the estimated parameters. Theoretically, anomaly or out-of-distribution (OOD) detection might also be addressed by additional evaluations, e.g., by analyzing the reconstruction loss of an autoencoder applied before the main data-driven model [4]. However, such techniques extend the inference time as a subsequent ML procedure is still required to provide the actual prediction.

Whereas in classical ML methods, network parameters ψ are solely trained to predict some target parameters θ from a feature set x, Bayesian inference, which is also called probabilistic or posterior inference, enables the estimation of a posterior distribution p(ψ|x,θ) of the network parameters given knowledge or assumptions, i.e., the prior, and observed data, i.e., the likelihood. Bayesian inference, therefore, offers a way to quantify the uncertainty of model outputs $\hat{\theta }$ [5]. Thus, when a ML model using Bayesian inference trained on a dataset ${\left\{(x^{\left(m\right)},\theta^{\left(m\right)})\right\}}_{m=1}^{M}$ with *M* samples shows large uncertainty in one of the output parameters during evaluation, on the one hand, this might indicate the need for more data in this parameter region. On the other hand, if after deployment during inference in final test large uncertainty is returned for a sample, this information could be used to make the decision to apply a physical model or some heuristic methods instead of using the output of the ML network for this specific device under test (DUT) to identify abnormal or OOD samples and to deal with those separately.

Therefore, it is preferable to apply a ML Bayesian inference method, which computes parameters precisely and with computational efficiency with reliable uncertainty estimates providing interpretability and auditability.

### 1.1. Parameter Extraction from Dynamic MEMS Tests

During testing of capacitive MEMS accelerometers, dynamic measurements can be carried out by applying electrical voltages to the capacitors through the application-specific integrated circuit (ASIC) [6,7]. When the target parameters are ill-conditioned, i.e., are not identifiable from electrical excitation only, these electrical tests are augmented by applying mechanical stimuli, e.g., ±1 g deflections.

The system response appearing as capacitive changes can then be analyzed with respect to the parameters of interest [1,7]. For capacitive MEMS accelerometers, these usually include the damping factor $D_{L}$, resonance frequency $f_{0}$, inertial mass *m*, and offset $d_{off}$ to the initial position of the mass. Moreover, the Brownian noise and the sensitivity are also often of interest [1], as well as process parameters such as the epitaxial layer thickness and edge loss.

Solving the underlying differential equations numerically becomes cumbersome when inhomogeneities and nonlinearities, e.g., originating from electrical force feedback of the ASIC, cannot be straightened out from the system differential equations [3].

### 1.2. Uncertainty in MEMS Testing

A variety of causes and influencing factors leads to uncertainties during the testing of MEMS devices. To a large extent they can be attributed to fabrication process variations [8,9,10,11]. Slight changes in material properties, for example, changes in the Young’s modulus of the polysilicon [9] or a change in the thermal conductivity [12] as well as material inhomogeneities such as varying grain size [13], can affect the etch processes, and therefore lead to differences in geometry, e.g., changing stiffness coefficients, gap distances, and capacitor areas. Furthermore, variation can be introduced by machine wear and aging [9], which does not only affect the tools in production, but also the measurement equipment, which might lead to different measurement errors depending on individual test benches. Moreover, generation-recombination, flicker, and Brownian noise [14], as well as the signal processing path of the sensor itself can lead to uncertainties in the estimation of system parameters. An example for the latter are low pass filters of the ASIC, which are applied for economic reasons and can also impede the distinction between actual changes in the signal and noise. Finally, approximation errors in numerical methods used for system identification especially in the presence of ill-conditioned parameters also provide sources of uncertainty.

The goal of this paper is to answer the question as to how to make parameter extraction from dynamic measurements in MEMS testing more robust by analyzing the uncertainty of neural network estimations. Four ML Bayesian inference architectures are investigated, focusing on two main aspects. The first objective is to compare the architectures regarding their general predictive performance. The second objective is to evaluate the reliability of the uncertainty estimates given by the four architectures within the training distribution as well as under OOD conditions. Thus, three scenarios are investigated using simulated turn-off transients of capacitive MEMS accelerometers, namely the available training set size, sudden increase of measurement noise, and a change in damping factor, e.g., due to leakage.

The paper is organized as follows. Section 2 briefly categorizes and summarizes related work on uncertainty quantification in the context of MEMS fabrication and testing. Section 3 introduces important concepts and methods for uncertainty quantification with neural networks including the relevant evaluation metrics. Section 4 describes the datasets used and provides details on the experimental procedure and the applied ML methods. In Section 5, the results are divided into the comparison of the ML methods on the synthetic data, evaluation on noisy data, and evaluation of systems with increased damping factor, which are subsequently discussed in Section 6. The conclusion is drawn in Section 7.

## 2. Related Work

The related work is a summary of uncertainty quantification in MEMS concerning applications. Extracting relevant system parameters from either electrically or mechanically excited MEMS tests during wafer-level testing (WLT) [7,15] as well as final testing (FT) [1] is targeted by various approaches, depending on the complexity of the underlying system. Whereas for some systems, the fitting of coefficients of an equation for sensitivity determination [1] provides sufficient precision, other systems require the application of multivariate adaptive regression splines (MARS) [16], building ensembles of MARS or support vector machines (SVMs) [2], or different variants of neural networks [3].

Most UQ related work in the application area of MEMS focuses on the analysis of uncertainty arising from material properties and geometrical changes studying the resulting effects on performance parameters. These include sensitivity studies based on Monte-Carlo simulations from finite element models analyzing uncertainty propagation [9], sensitivity due to uncertainty of diaphragm parameter [17], and the quantification of uncertainty originating from process variations via tensor recovery [11]. Furthermore, stochastic effects from the micro structure of polysilicon films [13], uncertainties in stiction [18], or uncertainty arising from creep failure [19] have been evaluated. The work whose objectives come closest to the work presented in this paper was made by Gennat et al. [20]. In the context of rapid optical testing of a MEMS resonator, through multivariable finite element analysis, a polynomial was developed describing the DUT-specific parameters mechanical stress, flexure thickness and flexure width dependent on eigenfrequencies measured during WLT, while providing uncertainty estimates for each parameter and DUT from the Chebyshev optimization. However, for the overdamped MEMS accelerometers targeted within the present paper, parameter identification relying on numerical solutions does not meet the strict time constraints and therefore the use of ML-driven approaches is required, for which uncertainty estimates need to be obtained in a different manner as described in Section 3.

Dealing with model selection for radio-frequency MEMS switches, Ling and Mahadevan [21] evaluated a general polynomial chaos surrogate model through classical and Bayesian hypothesis testing, reliability-based evaluation, and an area metric-based method, which compares prior and posterior distributions. Mullins et al. [22] built on this work and suggested a weighted evaluation of the different epistemic uncertainty estimates using a Gaussian Process (GP) as surrogate model.

Even though UQ methods for ML architectures have not gained a lot of attention in the area of MEMS production and testing as of yet, their use has been successfully demonstrated in other safety and time-critical applications. Auspicious examples are the application of MDNs in autonomous driving [23] and the use of bootstrapping and Monte-Carlo dropout for collision avoidance in robotics by controlling the movement speed according to uncertainty estimates [24]. Furthermore, BayesFlow was shown to reach encouraging accuracy in the parameter recovering on a macroeconomic agent-based model [25] and its use to infer spreading dynamics of diseases via Bayesian inference has been demonstrated [26].

## 3. Methods for Uncertainty Quantification

There are three categories of tasks which can be addressed by different methods for uncertainty quantification, summarized by Lust et al. [27]; First, in the setup of predictive uncertainty, mainly samples within the training distribution and thus within the generalization envelope of a deep neural network (DNN) are taken into account. Samples with larger uncertainty scores assigned are associated with a more error-prone output. The second objective is anomaly or OOD detection, i.e., the identification of whether an input sample belongs to the training distribution. The third objective is the security of the system by aiming to detect synthetically generated or manipulated inputs, which might be used to deliberately provoke wrong outputs, called adversarial examples.

In this work, the focus is on the first two tasks, where the distinction between them is based on the fact that the overall predictive variance or uncertainty of a prediction arises from two components, namely epistemic uncertainty and aleatoric uncertainty [28,29,30,31]. Epistemic uncertainty $\sigma_{e}^{2}$ is associated with the uncertainty which arises from the choice of network parameters and is due to phenomena unexplained by the ML model [28], and thus also called model uncertainty. The aleatoric uncertainty component $\sigma_{a}^{2}$ originates from noise in data, e.g., due to the measurement error of a test bench. The ML model cannot compensate for measurement error, thus this contribution cannot be reduced for example by gathering more data. The aleatoric component can be either homoscedastic if all samples are subjected to the same systematic noise, or heteroscedastic if the noise is input dependent [32]. Thus, for a dataset ${\left\{(x^{\left(m\right)},\;\theta^{\left(m\right)})\right\}}_{m=1}^{M}$ the total predictive variance results from the sum of the two uncertainty components [23,33]:

(1) $$E∥\theta -\hat{f}{\left(x\right)∥}^{2}=\sigma_{a}^{2}+\sigma_{e}^{2}.$$

For further information on the basics of decision making under uncertainty, and a background on Bayesian inference, the reader is referred to [23,29,34].

Gaussian Processes and Markov Chain Monte-Carlo (MCMC) algorithms are designed to capture the uncertainty of their predictions. However, their computational expense usually disqualifies them for near real-time applications for complex systems [5,29,35]. For uncertainty quantification (UQ) with DNNs, variational inference (VI)-based approaches are widely used to approximate the posterior probability distribution. Since DNNs do not contain any confidence representation, variability has to be introduced by either creating a distribution over networks or over their parameters. An example for VI is the ensembling of multiple trained models [29,33,36,37]. If the different models return similar outputs for an input sample, it is more likely that the input lies within the generalization area of the models. However, a distinction of the uncertainty source is not possible with this method [29]. Another VI-based approach is the application of Monte-Carlo Dropout during inference [38], i.e., passing the same input sample through the same DNN multiple times while randomly setting weights to zero, and thus creating a distribution over several predictions.

### 3.1. Network Architectures of BNN, MDN, PBNN and BayesFlow

#### 3.1.1. Bayesian Neural Networks

Instead of the deterministic weights, which are used in common DNNs, Bayesian neural networks (BNNs) learn probability distributions over their weights starting from given priors. However, there exists no universally valid approach on the choice of the priors even though Gaussian distributions with zero mean are often assumed.

As in all VI-based approaches, the posterior distribution for an input $x^{\left(m\right)}$

(2) $$p\left(\hat{\theta }|x^{\left(m\right)},x,\theta \right)=\int p\left(\theta |x^{\left(m\right)},\psi \right)p\left(\psi |x,\theta \right)d\psi$$

needs to be approximated as it cannot be determined analytically [29,38]. In BNNs, this is done by minimizing the Kullback–Leibler (KL) divergence $D_{KL}$ [39] between the true and estimated posterior distribution, here denoted as p(θ|x) and $p_{\psi }\left(\theta |x\right)$ with

(3) $$D_{KL}\left(p\left(\theta |x\right)∥p_{\psi }\left(\theta |x\right)\right)=\int p\left(\theta |x\right)\log\frac{p(\theta |x)}{p_{\psi }\left(\theta |x\right)}d\theta .$$

This is equivalent to minimizing the negative log-likelihood (NLL), i.e., performing maximum likelihood estimation (MLE) [28,40]. Although the true posterior distribution is unknown, minimizing the KL divergence enables the definition of the evidence lower bound (ELBO), which is a lower bound of the log marginal likelihood [28,41]. The ELBO can be maximized via stochastic variational inference (SVI), which enables the optimization via deep learning methods. During inference, multiple predictions of one input through network are conducted, sampling from the weight distributions multiple times. Thus, a non-parametric distribution is obtained [42]. The cost function is composed of a weighted sum of a term measuring how well the network fits the data $L_{\delta }\left(\theta ,\hat{\theta }\right)$ and the KL divergence for comparing the difference between the true and estimated posterior, which is multiplied by the KL weight $w_{KL}$:

(4) $$L=L_{\delta }\left(\theta ,\hat{\theta }\right)+w_{KL}D_{KL}\left(p\left(\theta |x\right)∥p_{\psi }\left(\theta |x\right)\right).$$

Within the training procedure, SVI can be implemented via the “Bayes-by-backpropagation” algorithm [42].

The computational effort can be reduced by using distributions over weights only in the last layer(s) [43]. However, a BNN constructed in the described way can only provide information regarding its epistemic uncertainty [42].

#### 3.1.2. Mixture Density Networks

An architecture which enables the prediction of parametric distributions and does not use sampling or probability distributions within the network is the mixture density network (MDN) [35]. It is trained to estimate the parameters of the posterior distribution, e.g., in the form of a Gaussian mixture model (GMM) within one prediction pass. Thus, in the case of a mixture of Gaussian distributions, $\theta_{\psi }={\left\{\mu_{j},\sigma_{j},\alpha_{j}\right\}}_{j=1}^{K}$ with mean or expected value $\mu_{j}$, standard deviation $\sigma_{j}$, and weight coefficients $\alpha_{j}$, $\sum_{j}\alpha_{j}=1$, for a predefined number of mixture components *K* [5]. In the case of a unimodal output, the weight coefficients are discarded. The total expectation E and variance V of a Gaussian mixture are given by the following equations [23,35,44]:

(5) $$E\left[\theta |x\right]=\sum_{j=1}^{K}\alpha_{j}\left(x\right)\mu_{j}\left(x\right),$$

(6) $$V\left[\theta |x\right]=\sum_{j=1}^{K}\alpha_{j}\left(x\right)\sigma_{j}\left(x\right)+\sum_{j=1}^{K}\alpha_{j}\left(x\right){∥\mu_{j}\left(x\right)-\sum_{k=1}^{K}\alpha_{k}\left(x\right)\mu_{k}\left(x\right)∥}^{2}.$$

Although any posterior can be approximated by a mixture of sufficient components, the MDN architecture is only able to capture aleatoric uncertainty as it does not provide variability within an individual prediction. Choi et al. [23] argued that when using more than one component, such a variability can though again be introduced. Additionally, the known difficulty of mode collapse in this architecture can lead to the disregard of modes in multimodal distributions [45].

#### 3.1.3. Probabilistic Bayesian Neural Networks

Probabilistic Bayesian neural networks (PBNN) are built up with the two network architectures described above, i.e., network weights are defined as distributions and a MDN output layer is attached to the network. Thus, each of *T* repeated prediction passes of an unchanged input vector leads to a slightly different parameterized posterior. In case of a mixture of Gaussian distributions, the total variance is calculated by applying Equation (6) with equal weights for each distribution. Furthermore, the mean over the standard deviations represents the data uncertainty $\sigma_{a}^{2}$ [23]. Thus, with $\sigma_{e}^{2}$ as residual, PBNNs are able to capture both aleatoric and epistemic uncertainty.

#### 3.1.4. BayesFlow

BayesFlow [46] is a Bayesian inference method in use of an invertible neural network. The invertible neural network is based on normalizing flows, which allow for inference from a simple probability density to a complex distribution through a series of invertible mappings [47]. The distribution inference with implicit form is directly obtained from the neural network, and thus can be very exact. The network is trained to learn a global estimator for the probabilistic mapping from observed data to underlying physical parameters, then the well-trained network can directly infer the posterior of physical parameters from the same physical model family.

**Figure 1:**
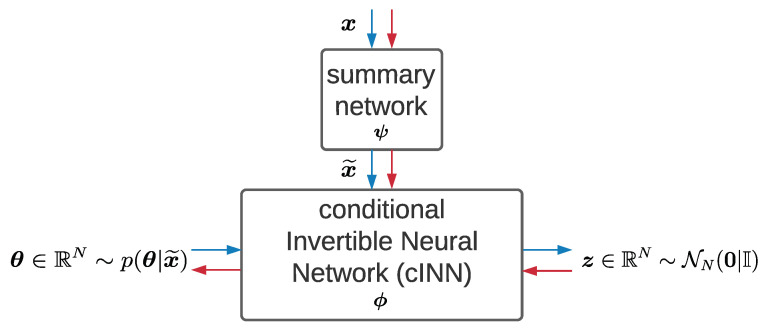
BayesFlow Architecture. ψ contains the network parameters of the summary network, ϕ the network parameters of the cINN. The blue arrows stand for the forward process; the red arrows stand for the inverse process.

The architecture of BayesFlow (shown in Figure 1) consists of a summary network to reduce the dimensionality of the observed data x and a conditional invertible neural network (cINN) to transform the distributions implicitly between the physical parameters θ and latent variables z. The network parameters of the summary network and cINN are denoted as ***ψ*** and ***ϕ***, respectively. Both parts can be optimized jointly via back propagation by minimizing the KL divergence between the true and the model induced posterior of θ. Then, the objective function can be written as follows:

(7) $$\hat{\phi },\hat{\psi }=\underset{\phi ,\psi }{\mathrm{argmin}}\;E_{p(x)}\left[\mathrm{KL}(p\left(\theta |x\right)|\right|p_{\phi ,\psi }\left(\theta |x\right)].$$

The summary network $h_{\psi }$ is supposed to be adjusted to the observed data x. For example, a long short-term memory network (LSTM) [48] is a typical architecture for time-series data. In this way, the compressed data $\tilde{x}$ with informative statistics can be passed through the cINN and taken as the condition while inducing the posterior of physical parameters θ, namely $p_{\phi }\left(\theta |\tilde{x}=h_{\psi }\left(x\right)\right)$.

The cINN, assumed as an invertible function $f_{\phi }$, is built up from a chain of conditional affine coupling blocks [49], the structure of which ensures the neural network to be invertible, bijective and to have easily calculable Jacobian determinant $J_{f_{\phi }}$ [50]. In the forward direction, the input is the physical parameters $\theta \in R^{N}$, while the output is the latent variables z, which follow a standard normal distribution $p\left(z\right)=N_{N}\left(z|0,I\right)$. Via the change-of-variables formula of probability, the posterior can be reformulated as

(8) $$p_{\phi }\left(\theta |\tilde{x}\right)=p\left(z=f_{\phi }\left(\theta ;\tilde{x}\right)\right)\left|\det J_{f_{\phi }}\right|.$$

For a batch of dataset ${\left\{(x^{\left(m\right)},\theta^{\left(m\right)})\right\}}_{m=1}^{M}$, the objective function becomes

(9) $$\hat{\phi },\hat{\psi }=\underset{\phi ,\psi }{\mathrm{argmin}}\;\frac{1}{M}\sum_{m=1}^{M}(\frac{{∥f_{\phi }\left(\theta^{\left(m\right)};h_{\psi }\left(x^{\left(m\right)}\right)\right)∥}^{2}}{2}-\log|\det J_{f_{\phi }}^{\left(m\right)}\left|\right).$$

In the inverse direction, the physical parameters θ can be obtained by calculating the mean of the posterior $p_{\phi }\left(\theta |\tilde{x}\right)$ with the well-trained network and sampled variables z. For an observation of test x with sampling a latent variable *T* times, this process can be formulated as follows:

(10) $${\hat{\theta }}^{\left(m\right)}=\frac{1}{T}\sum_{t=1}^{T}f_{\hat{\phi }}^{-1}\left(z^{\left(t\right)};h_{\hat{\psi }}\left(x^{\left(m\right)}\right)\right).$$

### 3.2. Metrics

To evaluate the quality of Bayesian inference, on the one hand, the accuracy, precision, and reliability of the estimation of model parameters are taken into consideration. On the other hand, the posterior distribution of model parameters is investigated, regarding uncertainty, consistency with the prior distribution, and multicollinearity between model parameters.

1. In terms of the regression accuracy between the estimation and the ground truth of the model parameters, normalized root mean squared error (NRMSE) and coefficient of determination ($R^{2}$) are two standard metrics. Moreover, in practice it is also important to have information about the maximum absolute error (MAXAE) and mean absolute error (MAE). For a group of estimated ${\left\{{\hat{\theta }}^{\left(m\right)}\right\}}_{m=1}^{M}$ and true parameters ${\left\{\theta^{\left(m\right)}\right\}}_{m=1}^{M}$ with the mean of the true parameters $\bar{\theta }$, these metrics can be calculated as follows:
  (11) $$NRMSE=\frac{\sqrt{\frac{1}{M}\sum_{m=1}^{M}{\left(\theta^{\left(m\right)}-{\hat{\theta }}^{\left(m\right)}\right)}^{2}}}{\theta_{max}-\theta_{min}},$$
  (12) $$R^{2}=1-\sum_{m=1}^{M}\frac{{\left(\theta^{\left(m\right)}-{\hat{\theta }}^{\left(m\right)}\right)}^{2}}{{\left(\theta^{\left(m\right)}-\bar{\theta }\right)}^{2}},$$
  (13) $$MAXAE=\max_{m=1,...,M}\left|\theta^{\left(m\right)}-{\hat{\theta }}^{\left(m\right)}\right|,$$
  (14) $$MAE=\frac{1}{M}\sum_{m=1}^{M}\left|\theta^{\left(m\right)}-{\hat{\theta }}^{\left(m\right)}\right|.$$
2. In terms of the precision and reliability of the estimation, the normalized mean confidence interval width (NMCIW) and confidence interval coverage probability (CICP) at the 95% confidence level are assessed. The higher the CICP is, the more reliable the estimation could be. Whereas the smaller the NMCIW is, the more precise the estimation could be. For the *m*-th parameter by sampling it *T* times, the standard deviation is $\sigma_{{\hat{\theta }}^{\left(m\right)}}$, the 95% confidence interval (CI) with lower limit $L_{m}$ and upper limit $U_{m}$ is
  (15) $$CI^{\left(m\right)}\left(95\%\right)=\left[L_{m},U_{m}\right]={\hat{\theta }}^{\left(m\right)}\pm 1.96\sigma_{{\hat{\theta }}^{\left(m\right)}}.$$
  Then, the NMCIW and CICP for the entire group of parameters are
  (16) $$\begin{array}{c} CICP=\frac{1}{M}\sum_{m=1}^{M}c_{m},\;\mathrm{with}\;c_{m}=\left\{\begin{array}{cc} 0 & \;\mathrm{if}\;\theta^{\left(m\right)}\notin \left[L_{m},U_{m}\right],\\ 1 & \;\mathrm{otherwise}.\; \end{array}\right., \end{array}$$
  (17) $$NMCIW=\frac{1}{M}\sum_{m=1}^{M}\frac{U_{m}-L_{m}}{\theta_{max}-\theta_{min}}.$$
3. In terms of the uncertainty of posterior distribution, the negative log-likelihood (NLL) is calculated by assuming the posterior to be Gaussian distributed, which is guaranteed for MDN and PBNN. When the mean is taken to be the Gaussian NLL of the *M* data samples, the NLL is
  (18) $$\frac{1}{M}\sum_{m=1}^{M}\frac{1}{2}\left(\log\sigma_{{\hat{\theta }}^{\left(m\right)}}^{2}+\frac{{\left({\hat{\theta }}^{\left(m\right)}-\theta^{\left(m\right)}\right)}^{2}}{\sigma_{{\hat{\theta }}^{\left(m\right)}}^{2}}\right)+\frac{1}{2}\log\left(2\pi \right).$$

## 4. Experiments

### 4.1. Data Sets and Preprocessing

A high-granularity ASIC-MEMS reduced order model (ROM) of a capacitive MEMS accelerometer, which was described in [3], served as the basis for simulating 500 turn-off transients followed by the system response to a ±1 g excitation in a Monte-Carlo approach. The damping factor $D_{L}$, resonance frequency $f_{0}$, inertial mass *m*, offset $d_{off}$, as well as two process parameters $p_{1,2}$, served as labels. The dataset was partitioned into 200 simulated devices for training, 50 for validation, and 50 samples were retained for testing, thus never presented to the ML models during training and hyper parameter optimization. Additionally, the time series with a total number of 384 steps consisted of three non-continuous turning points, and thus were difficult to handle for the NNs. Therefore, they were split up into four segments with equal length. These are provided to the ML models as four input channels such that the dimension of the time series with 384 × 1 is changed to 96 × 4.

**Figure 2:**
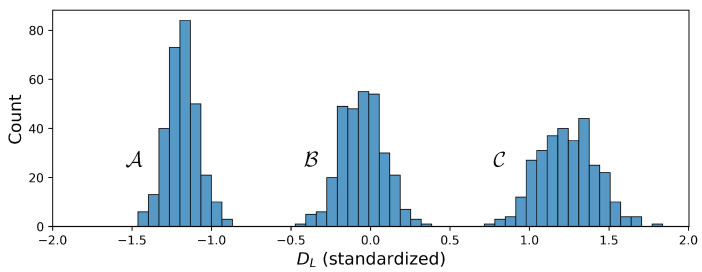
$D_{L}$ conditions denoted as A, B, and C.

In particular, to evaluate the model’s performance and its uncertainty scores on noisy time series, the training set was combined with white noise with amplitudes of 0.01, 0.025, and 0.05. Furthermore, the complete test set was perturbed with each of the noise variants for the regarding evaluation. The results are shown in Section 5.3. In addition, 300 devices were simulated around two higher damping factors, in the following denoted as condition B and C as shown in Figure 2. With these samples, two types of OOD experiments were performed. First, the models were trained on 200 samples and validated on 50 samples randomly drawn from all three distributions and subsequently evaluated on 50 additional samples, also drawn from all three distributions. Second, the models were trained only on 200 samples from the lowest $D_{L}$ distribution A and separately evaluated on 50 samples from each of the three distributions. The respective results are given in Section 5.4. Data from condition A was used in the other experiments. The preprocessing of data included standardization of the labels and scaling of the time series.

### 4.2. Implementation and Training of ML Models

All models were implemented in PyTorch [51]. A residual neural network (ResNet) [52] served as a reference for the predictive performance. The BNN, MDN, and PBNN were built on the ResNet architecture. For the BNNs, the implementation was based on [53], for the MDNs on [54]. Unless specified otherwise, the weights of the BNNs and PBNNs were initialized with zero-mean Gaussian priors, a KL weight $w_{KL}$ of 0.1 was used, and training was performed using the Adam optimizer. Early stopping was applied to prevent overfitting and for all models except for BayesFlow a dropout rate of 0.2 was used throughout the training process. Additional hyper parameters such as the number of neurons and layers, the learning rate and scheduler, as well as the stopping iteration, were optimized via Bayesian optimization [55]. The optimized BNN had 389,068 trainable parameters including mean and standard deviation for each weight, a fixed learning rate of $3\times 10^{-4}$ and a stopping iteration of 200 epochs. The MDN had 349,389 trainable parameters, an unvaried learning rate of $3\times 10^{-4}$ and early stopping was applied after 150 epochs. 487,945 trainable parameters were used for the optimized PBNN with a fixed learning rate of $10^{-4}$ and with a stopping iteration of 150. For BayesFlow, the optimization resulted in the use of 416,176 trainable parameters, a start learning rate lr of 0.001 and an exponential learning rate scheduler with $lr_{epoch}=0.5^{\left(epoch/2000\right)}lr_{\left(epoch-1\right)}$. During the uncertainty evaluation of the BNNs, PBNNs, and BayesFlow, 100 prediction passes were carried out for each sample. All metrics and plots are reported on standardized labels.

## 5. Comparison and Evaluation of the UQ Methods

The performance evaluations of the architectures are divided into the evaluation on simulated MEMS devices, on varied training set size, on noisy test sets and on higher damping factors. Especially, the epistemic and aleatoric uncertainty under those circumstances are analyzed with the PBNN, as it is impossible to perform this uncertainty decomposition from the other three architectures without major changes.

### 5.1. Evaluation on Simulated MEMS Devices

**Figure 3:**
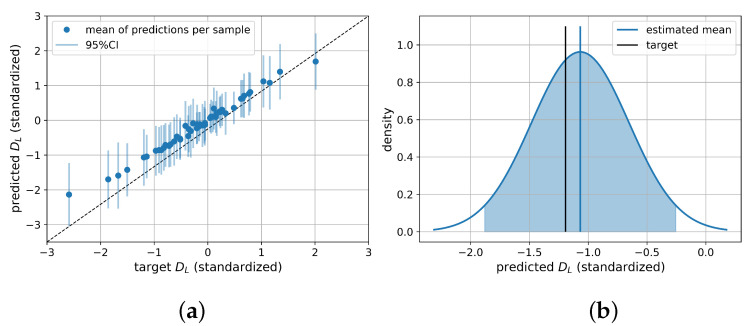
Performance of ResNet PBNN on test set for $D_{L}$; (**a**) scatter plot with 95%CI as error bars; (**b**) density plot for test observations composed of 100 evaluations of one sample. The highlighted area represents the 95%CI, the dashed line the target, the solid line the mean estimate.

All four uncertainty representing methods increased the predictive performance compared to a pure ResNet with an average NRMSE over all six parameters of 0.0831 on the test set. The MDN achieved an average NRMSE of 0.0424. BNN and PBNN showed similar results with an average NRMSE of 0.0278, and 0.0254, respectively. Figure 3a shows the predictions of the PBNN on the test set including the 95%CI calculated from the total variance for each sample. In Figure 3b, the composition of the 95%CI from the distributions given from 100 prediction passes for a single sample is shown. The BayesFlow architecture was able to capture the underlying relations best with an average NRMSE of 0.0112. A breakdown by the individual parameters is given in Table 1. Additional performance metrics on the test set, namely the $R^{2}$, MAE, and MAXAE can be found in Appendix A.

Furthermore, differences became visible in the comparison of the NMCIWs and CICP scores on the test set, which are given in Table 2. Whereas BayesFlow returned narrow intervals with an average NMCIW over all parameters of 0.0417, the NMCIW of the MDN was 0.146, for the BNN 0.211, and for the PBNN 0.272. Accordingly, the CICP of the PBNN was always higher than that of the BayesFlow model.

**Figure 4:**
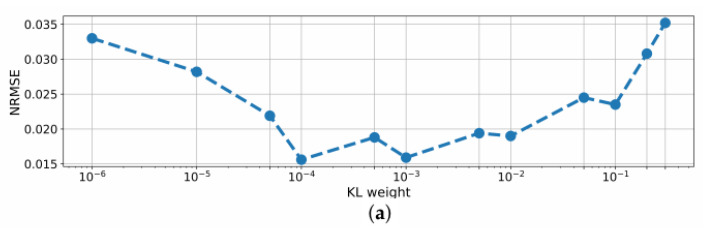

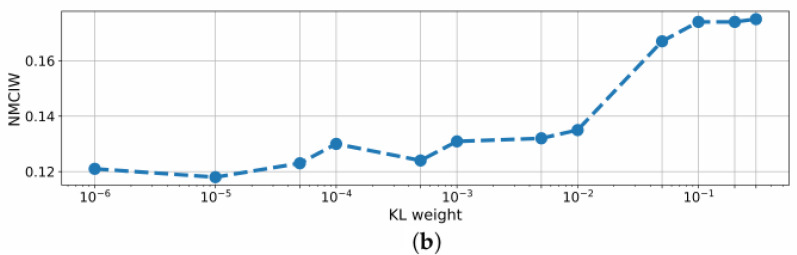
(**a**) NRMSE and (**b**) NMCIW for BNN prediction of $D_{L}$ on the test set using different KL weights during training.

A variation of the KL weight $w_{KL}$ in the BNN led to strong changes in performance and quality of uncertainty scores, which are shown in Figure 4.

On the one hand, the NRMSE of all parameters was reduced for $10^{-4}\leq w_{KL}\leq 10^{-2}$ with a minimum of 0.0156 at $w_{KL}$ of $10^{-4}$. Smaller values for the KL weight, however, led to a performance deterioration. On the other hand, the NMCIW decreased with lower KL weights and the uncertainty scores of the network did not reveal meaningful changes for the OOD conditions reported in the next sections. A change in the standard deviation of the priors led to a decrease in performance and deteriorated the training process.

**Figure 5:**
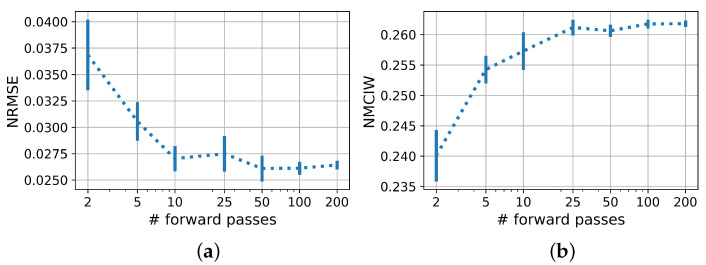
(**a**) NRMSE and (**b**) NMCIW of PBNN in prediction of $D_{L}$ with varied number of forward passes. Error bars show the standard deviation over 10 iterations.

Figure 5 shows the influence of the number of predictions for each test sample carried out with a PBNN for each sample on NRMSE and NMCIW. For up to 50 evaluations, NRMSE and NMCIW showed large deviations when the experiment was repeated 10 times, which flattened with more evaluations.

**Figure 6:**
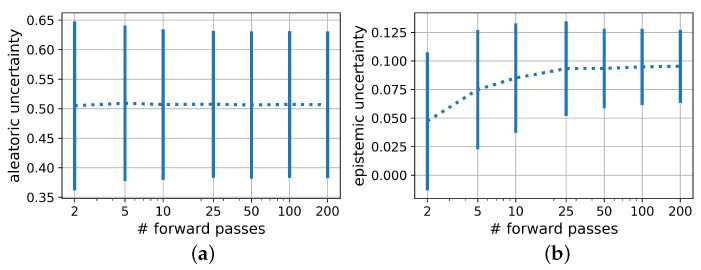
(**a**) Aleatoric and (**b**) epistemic uncertainty with number of prediction passes. Error bars show the average over standard deviations.

As visualized in Figure 6, the variance in estimating the epistemic uncertainty also decreased with the number of evaluations. Mean and standard deviation of the aleatoric uncertainty, however, remained mostly unaffected by the number of evaluations per sample.

### 5.2. Influence of Varied Training Set Size

The NRMSE as well as the NMCIW of all models increased upon halving the training dataset as shown in Table 3. Accordingly, these metrics decreased upon doubling the number of training samples in all models but the BNN. For reference, the NRMSE of the deterministic ResNet was 0.132 and 0.0622 for $D_{L}$ with 100 and 400 training samples, respectively. For the MDN, the ratio of samples, for which the true parameters were not captured by the CI, decreased especially noticeably with less training data, whereas, in contrast, the CICP of BayesFlow dropped to 0.147 with 400 training samples.

**Figure 7:**
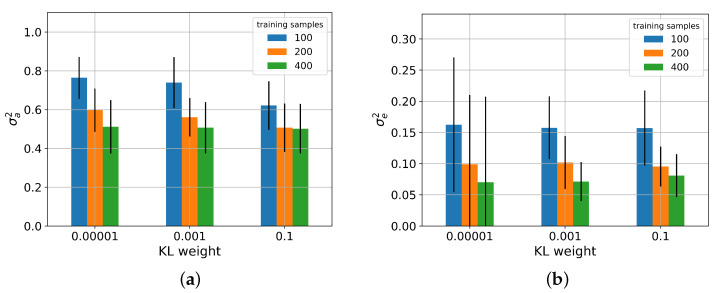
Aleatoric (**a**) and epistemic (**b**) uncertainty components of PBNN under variation of the training set size and the KL weight.

When decomposing epistemic and aleatoric uncertainty components during the prediction of $D_{L}$ with the PBNN as shown in Figure 7, both components decreased with larger training set sizes. On all three datasets, the aleatoric uncertainty had the greater share of the overall uncertainty. However, the aleatoric uncertainty decreased with higher KL weights, whereas the epistemic uncertainty remained unaffected. Additionally, a visible decrease between the uncertainty scores for the model trained on only 100 samples compared to a training set size of 200 and 400 became apparent. The difference between the aleatoric uncertainty predictions when trained on 200 and 400 samples was reduced with increasing KL weight.

### 5.3. Performance on Noisy Data

**Figure 8:**
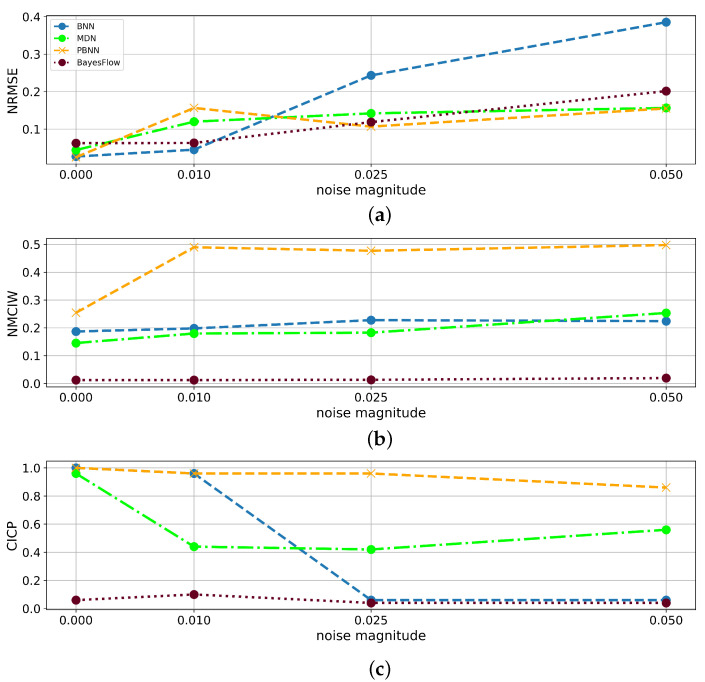
(**a**) NRMSE, (**b**) NMCIW, and (**c**) CICP plotted for each magnitude of white noise added to the test set in the prediction of $D_{L}$. All noise variants were included in the training set.

Predictions on input signals disturbed by varying levels of noise led to performance deterioration of all networks as shown in Figure 8. In particular, the NRMSE of the BNN increased considerably stronger than the NRMSE of the other architectures. The NMCIW revealed further differences: whereas the interval widths of the BayesFlow model and the BNN remained at a constant level, the NMCIW of the MDN increased monotonously over the applied noise magnitudes. For the PBNN, the NMCIW almost doubled in the presence of noise, but the noise magnitudes were indistinguishable by the size of the NMCIW. Consequently, the CICP of BayesFlow was constantly very low, the CICP of the PBNN constantly high and slightly decreasing for the highest noise amplitude. The CICP of the MDN dropped at the lowest noise amplitude to around 0.4, the CICP of the BNN dropped at a noise magnitude of 0.025 to the level of BayesFlow.

Table 4 summarizes the effect of the noise magnitude on the uncertainty components identified by the PBNN. Comparison of the uncertainty components shows that the epistemic uncertainty comprised about 18% on clean and 5.9% to 8.6% on noisy inputs. Whereas for increased noise levels, the epistemic uncertainty of the PBNN constantly remained around a mean of 0.0854, a sharp increase between the aleatoric uncertainty on clean and noisy samples was observed even if no clear distinction between the noise levels was possible based on the returned aleatoric uncertainty scores.

### 5.4. Performance on Higher Damping Factors

The NRMSE, NMCIW, and CICP of networks trained on samples from all three damping areas are reported in Table 5. The total uncertainty of the PBNN for $D_{L}$ was 0.503 with an aleatoric share of 90.5%.

**Figure 9:**
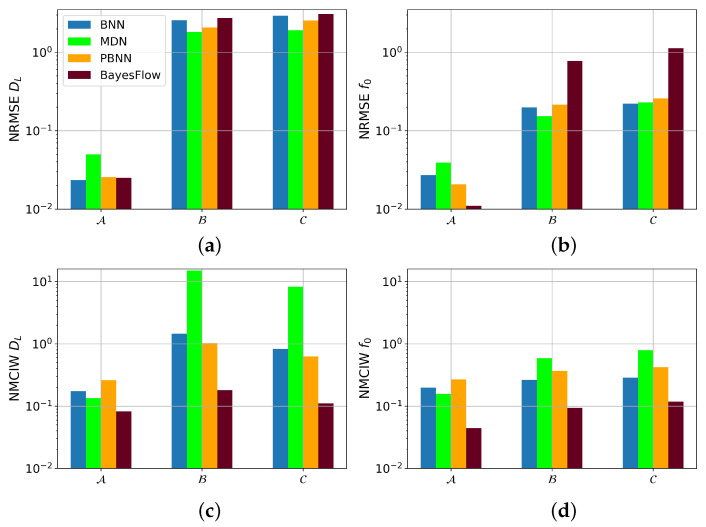
NMCIW and NRMSE in prediction of $D_{L}$ and $f_{0}$ with model trained only on samples from condition A. A, B, and C denote the three distributions over the true $D_{L}$ with increasing means. (**a**) NRMSE for $D_{L}$, (**b**) NRMSE for $f_{0}$, (**c**) NMCIW for $D_{L}$, (**d**) NMICW for $f_{0}$.

NRMSE and NMCIW for the prediction of $D_{L}$ and $f_{0}$ with the four architectures subsequently applied to the three damping modes after the networks have only been trained on the lowest damping mode, here denoted as A, are visualized in Figure 9. The predictive performance of all four models diminished for the OOD conditions. In the prediction of $D_{L}$, the MDN had the lowest NRMSE of the four models with 1.82 and 1.91 for B and C, respectively. The NRMSE of the BNN was 2.57 for the predictions on B and 2.93 for the samples from C, the NRMSE of the PBNN decreased to 2.08 and 2.55, and the BayesFlow model showed the largest NRMSE with 2.73 and 3.09 for B and C, respectively. The NMCIWs of all architectures were larger for $D_{L}$ than for the other parameters on OOD samples, even though the NMCIW did not increase with the distance from the training distribution as was the case for the other five parameters.

### 5.5. Overall Comparison of Predictive Performance and Uncertainty Estimates

**Figure 10:**
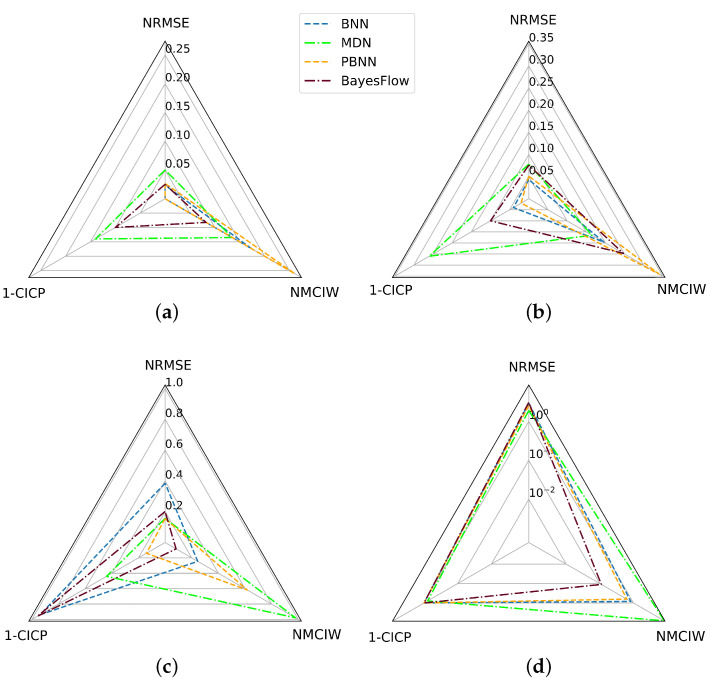
Radar charts summarizing the findings on the test sets with respect to the predictive performance and consistency of uncertainty estimates for the prediction of $D_{L}$ of BNN, MDN, PBNN, and BayesFlow. (**a**) 200 training samples, (b) 100 training samples, (c) 200 noisy training samples, noise amplitude of 0.05 in test set, (d) 200 training samples from damping condition A evaluated on C with logarithmically scaled axes.

Figure 10 compares the findings for the prediction of $D_{L}$ with respect to the predictive performance in terms of the NRMSE and the consistency of the uncertainty estimates in terms of the NRMSE and 1-CICP value for the BNN, MDN, PBNN, and BayesFlow. Based on the areas covered by the triangles arising from the three metrics visualized in the radar charts, in Table 6, a ranking of the four methods is derived for the four train-test conditions, juxtaposed in Figure 10. In this case, the networks were only trained and tested on clean training and test data originating from the same distribution, the resulting area between the metrics was lowest for the BNN, followed in decreasing order by the PBNN, BayesFlow, and MDN. For models trained on 200 variously noisy training samples and tested on samples with a noise amplitude of 0.05, the area was smallest for the PBNN, closely followed by the BayesFlow model. The later showed the smallest area of all models trained on 200 samples from damping condition A and evaluated on DUTs from condition C.

## 6. Discussion

All uncertainty expressing methods showed an increase in the overall performance compared to a pure ResNet architecture. This effect was reported by other works as well, e.g., [56,57], especially for OOD samples and small datasets. By taking the probabilistic perspective, averaging predictions drawn from continuous distributions resembles the usage of an infinite ensemble, which explains the improvement in performance.

The predictive performance of the BayesFlow architecture, however, was considerably higher than that of BNN, MDN, and PBNN on the same amount of training data. After doubling the training set size, the performance of the latter three architectures, however, improved, but they still did not outperform the BayesFlow network on average. The reason for this is twofold: First, the separate summary network of the BayesFlow architecture, which focuses on learning the representation of observed data, is optimized jointly with the inference network, which learns the posterior distribution of physical parameters. This separation of architecture encourages the learned representation to be as improving as possible for the inference of parameters’ distribution. Second, BayesFlow generates samples of posterior distribution of physical parameters according to the well-trained network without assuming its shape. This implicit network-induced posterior contributes to the more precise estimation of physical parameters. Another difference between the architectures was the quality of their uncertainty scores. In BayesFlow and PBNN, parameters with small predictive errors such as $d_{off}$ were predicted with smaller intervals. For BNN and MDN this was not the case, thus, the latter two architectures require the definition of a parameter individual threshold for OOD detection.

Furthermore, in all experiments, BayesFlow gave the narrowest intervals and, associated therewith, the lowest CICP values, thus overestimating its predictive performance or underestimating the underlying uncertainties. On the contrary, the NMCIW of the MDN was often the largest. Despite that, the CICP of the MDN was often smaller than 0.95, even on the unperturbed test set. This can be explained by the general lower performance of the MDN compared to BNN, BayesFlow, and BPNN with at the same time underestimated uncertainty. Overall, the PBNN led to the best CICP scores, which might be due to its capability of capturing both aleatoric and epistemic uncertainty components. For the remaining architectures, calibration of the uncertainty scores on a separate calibration dataset might prove useful [58,59].

The influence analysis of the number of evaluations for one input signal showed that for a sound statement on the predictive uncertainty at least 25, or better still, 50 to 200 evaluations were required. The NRMSE also benefited from an increased number of evaluations, analogous to ensemble methods. This should be considered when assessing the suitability of the models for deployment.

For the BNN, another adjusting option for performance improvement was provided by the KL weight $w_{KL}$. At a weight of $10^{-4}$, the BNN even outperformed BayesFlow regarding the NRMSE, however, the NMCIW did not reliably reflect the uncertainty anymore as the share of the KL loss in the cost function then becomes negligible. However, decreasing $w_{KL}$ led to a further drop in performance as the training stimulus to optimize the distributions over weights vanishes. In other works, $10^{-2}\leq w_{KL}\leq 10^{-4}$ was also found to be optimal [60]. Thus, a trade-off has to be accepted between predictive performance and quality of uncertainty score.

This becomes even more relevant when investigating the influence of the KL weight on the distinction between aleatoric and epistemic uncertainty in PBNNs, as shown in Figure 7. As expected, the epistemic uncertainty decreased with the doubling of the training set size. Counterintuitively, especially for low KL weights, the aleatoric uncertainty also decreased with larger training set sizes. This might be explained by the poor generalization of the model trained on a too small dataset reflected by the comparatively high epistemic uncertainty. The effect is therefore expected to vanish for larger datasets. Huseljic et al. postulated that high epistemic uncertainty entails high aleatoric uncertainty [61]. Thus, even for a KL weight of 0.1, the difference in the aleatoric uncertainties between the model trained on 100 samples compared to the ones trained on 200 and 400 samples remained visible even if the difference between the latter two equalized as expected. In practice, if very few training samples are available and the model uncertainty is high, the distinction between the two uncertainty components therefore has to be regarded with special caution.

Furthermore, a relation between KL weight and returned aleatoric uncertainty appeared for 100 and 200 training samples. For 400 training samples, the KL weight did not influence the aleatoric uncertainty anymore, indicating that here the number of training samples was sufficient to reduce both loss terms irrespective of their weighting.

In the presence of noisy inputs, as expected, the epistemic uncertainty given by the PBNN remained on a constant level, as noisy time series were already contained in the training dataset, and the aleatoric uncertainty sharply rose for disturbed inputs. This matches the observation that the NMICW of the MDN increased strictly monotonously with the noise amplitude whereas BNN and BayesFlow, unable to capture aleatoric uncertainty, did not show an increase of the NMICW resulting in low CICP values. For the PBNN, a notable step between clean and noisy data was visible, making it the only model with a high CICP even for noisy inputs. The increased epistemic uncertainty when trained on samples with varying noise levels compared to clean data is plausible as the model thereby was trained on less data for each individual noise domain. During all experiments, only homoscedastic noise has been applied, which might in practice not always be the case, e.g., due to influences from measurement equipment or ASIC. Additionally, it might be interesting for the application in FT to further split the detected data uncertainty into the individual sources described in Section 1.2.

It is well-known from the literature that purely data-driven models usually do not perform well outside of their training distribution. Thus, as expected, the NRMSE in the prediction of $D_{L}$ from the test set containing DUTs with higher damping factors than the training set decreased compared to models trained on samples from all three distributions, e.g., from 0.0391 to 1.82 for B and 1.91 for C for the BNN. Process drifts and defects, however, are commonly observed from productive data, which makes it important to closely evaluate how the confidence interval widths of the individual architectures reflect their uncertainty. From the OOD-experiments with DUTs with damping factors outside of the training distribution, it became visible, that the uncertainty intervals of BNN, MDN, and PBNN increased steeply for these test samples. However, similar to the observations for the incremental noise magnitudes, the predictive error of the BayesFlow model, which by far outperformed the other architectures on samples within the training distribution, heavily increased for DUTs with higher damping factors, but the interval widths did not reflect this behavior as distinctly as the other architectures. As the BayesFlow network also showed the worst generalization performance for the prediction of the resonance frequency for DUTs with previously unseen damping factors and gave low CICP scores for the other experiments, one might conclude that the BayesFlow model constituting the most complex one of the evaluated network architectures, slightly overfitted during the training process. This hypothesis is supported by the tremendous influence of the training set size on the NMCIW and CICP of BayesFlow. It could be interesting to investigate whether countermeasures to overfitting might also increase the CICP of BayesFlow models, for samples outside of the training distribution. To increase the confidence interval and improve the robustness of BayesFlow, some stochastic components should be extended in the future work.

## 7. Conclusions

Even if methods for uncertainty quantification in NNs have a strong theoretical foundation and provide the opportunity to augment the expressive power of network predictions without much overhead, they are rarely used in the context of manufacturing and MEMS testing in particular. As a wide variety of methods exist, this paper sought to identify a suitable approach based on Bayesian inference for the task of system identification from turn-off transients of capacitive MEMS accelerometers during FT. It was shown that the considered uncertainty representing methods did not only provide error estimates for individual predictions, but also increased the overall predictive performance. In particular, this applies to the BayesFlow architecture, which outperformed the other considered architectures in almost all experiments. Despite its high overall predictive performance, the BayesFlow model overestimated its predictions resulting in too small uncertainty scores requiring subsequent calibration. Thus, the choice of the specific architecture depends on whether priority is given to an excellent performance or an increased interpretability. For the BNN, this trade-off became apparent in the choice for the KL weight hyper parameter. If the goal is to accurately distinguish between uncertainty components aiming to increase the understanding of the model, PBNNs have been proved useful. As the epistemic uncertainty usually only had small shares of one up to ten percent of the total uncertainty, the specific uncertainty composition might be analyzed before deployment on test cases such as shown above with a large number of prediction passes. Thereafter, it might be sufficient to only capture aleatoric uncertainty during testing to decide whether to trust the NN output or fall back on numerical ODE solutions. For future research on the parameter identification from dynamic MEMS tests, it might be interesting to combine UQ approaches with physics-informed NNs as, e.g., suggested by Yang et al. [62].

## Figures and Tables

**Table 1:** NRMSE on test set. Lower is better.

	ResNet	BNN	MDN	PBNN	BayesFlow
$D_{L}$	0.0893	**0.0235**	0.0500	0.0264	0.0251
$f_{0}$	0.0883	0.0273	0.0394	0.0207	**0.0111**
*m*	0.102	0.0286	0.0486	0.0280	**0.0100**
$d_{off}$	0.047	0.0269	0.0269	0.0260	**0.00116**
$p_{1}$	0.087	0.0332	0.0501	0.0300	**0.00870**
$p_{2}$	0.085	0.0275	0.0394	0.0214	**0.0111**
${\bar{NRMSE}}_{test}$	0.0831	0.0278	0.0424	0.0254	**0.0112**

**Table 2:** NMCIW and CICP on test set.

		BNN	MDN	PBNN	BayesFlow
NMCIW	$D_{L}$	0.174	0.135	0.261	0.0823
	$f_{0}$	0.199	0.157	0.269	0.0442
	*m*	0.182	0.140	0.285	0.0405
	$d_{off}$	0.234	0.145	0.252	0.00237
	$p_{1}$	0.179	0.141	0.292	0.0363
	$p_{2}$	0.198	0.156	0.270	0.0444
CICP	$D_{L}$	1.0	0.86	1.0	0.90
	$f_{0}$	1.0	0.94	1.0	0.90
	*m*	0.98	0.84	1.0	0.90
	$d_{off}$	1.0	1.0	1.0	0.90
	$p_{1}$	0.98	0.86	1.0	0.96
	$p_{2}$	1.0	0.94	1.0	0.90

**Table 3:** NRMSE, NMCIW, and CICP for the prediction of $D_{L}$ on the test set with models trained on 100, 200, and 400 samples.

Training Samples		BNN	MDN	PBNN	BayesFlow
	NRMSE	0.0431	0.0779	0.0511	0.0758
100	NMCIW	0.196	0.165	0.340	0.247
	CICP	0.96	0.74	0.98	0.90
	NRMSE	0.0235	0.0500	0.0264	0.0251
200	NMCIW	0.174	0.135	0.261	0.0823
	CICP	1.0	0.86	1.0	0.90
	NRMSE	0.0242	0.0328	0.0228	0.0114
400	NMCIW	0.190	0.122	0.243	0.00332
	CICP	1.0	0.98	1.0	0.147

**Table 4:** Aleatoric and epistemic uncertainty of the PBNN in the prediction of $D_{L}$ from perturbed time series with the PBNN trained on samples from all noise domains.

Noise Magnitude	0.0	0.01	0.025	0.05
$\sigma_{a}^{2}$	0.479	1.028	1.032	1.069
SD	0.108	0.104	0.103	0.134
perc. of V[θ|x]	82.0%	91.4%	94.1%	93.5%
$\sigma_{e}^{2}$	0.105	0.0968	0.0650	0.0746
SD	0.0387	0.0291	0.0261	0.0194
perc. of V[θ|x]	18.0%	8.6%	5.9%	6.5%
V[θ|x]	0.584	1.125	1.097	1.1436

**Table 5:** NRMSE, NMCIW, and CICP are reported on test samples for BNN, MDN, PBNN, and a BayesFlow model trained on samples from all three $D_{L}$ modes A, B and C. The metrics evaluated on the test set are given for $D_{L}$ and the average over the parameters $f_{0}$, *m*, $d_{off}$, $p_{1}$ and $p_{2}$, which were not subject to a shift in the distribution.

Metric	Parameter (s)	BNN	MDN	PBNN	BayesFlow
NRMSE	$D_{L}$	0.0391	0.0572	0.0391	0.0134
NRMSE	all w/o $D_{L}$	0.0511	0.110	0.0817	0.0215
NMCIW	$D_{L}$	0.249	0.143	0.357	0.0388
NMCIW	all w/o $D_{L}$	0.193	0.299	0.346	0.0635
CICP	$D_{L}$	1	1	1	0.80
CICP	all w/o $D_{L}$	0.92	0.88	0.95	0.79

**Table 6:** Ranking of methods based on the area covered by the triangles built from the NRMSE, NMCIW, and 1-CICP values in the radar charts shown in Figure 10. Smaller areas are considered superior.

	Condition	100 Training Samples	200 Training Samples	200 Noisy Training Samples, Noise Amplitude of 0.05 in Test Set	200 Training Samples from Damping Condition A Evaluated on C
Method					
BNN		**0.00177**	**0.00780**	0.297	2.69
MDN		0.0141	0.0329	0.283	10.3
PBNN		0.00298	0.0109	**0.0882**	2.07
BayesFlow		0.00554	0.0221	0.125	**1.54**

## Data Availability

Not applicable.

## Abbreviations

The following abbreviations are used in this manuscript:

ASIC	application-specific integrated circuit
BNN	Bayesian neural network
CI	confidence interval
CICP	confidence interval coverage probability
cINN	conditional invertible neural network
DNN	deep neural network
DUT	device under test
ELBO	evidence lower bound
FT	final test
GMM	Gaussian mixture model
GP	Gaussian Process
KL	Kullback–Leibler
LSTM	long short-term memory network
MAE	mean absolute error
MARS	multivariate adaptive regression splines
MAXAE	maximum absolute error
MCMC	Markov Chain Monte-Carlo
MDN	mixture density network
MEMS	micro-electro-mechanical system
ML	machine learning
MLE	maximum likelihood estimation
NLL	negative log-likelihood
NMCIW	normalized mean confidence interval width
NN	neural network
NRMSE	normalized root mean squared error
ODE	ordinary differential Equation
OOD	out-of-distribution
PBNN	probabilistic Bayesian neural network
ROM	reduced order model
SVI	stochastic variational inference
SVM	support vector machine
UQ	uncertainty quantification
VI	variational inference
WLT	wafer-level test

## Appendix A

**Table A1:** $R^{2}$ on test set. Larger is better.

	ResNet	BNN	MDN	PBNN	BayesFlow
$D_{L}$	0.754	**0.983**	0.923	0.979	0.981
$f_{0}$	0.866	0.987	0.973	0.993	**0.998**
*m*	0.694	0.976	0.931	0.977	**0.997**
$d_{off}$	0.988	0.988	0.987	0.988	**1**
$p_{1}$	0.821	0.974	0.940	0.979	**0.998**
$p_{2}$	0.876	0.987	0.973	0.992	**0.998**

**Table A2:** MAE on test set. Smaller is better.

	ResNet	BNN	MDN	PBNN	BayesFlow
$D_{L}$	0.397	**0.0802**	0.147	0.0865	0.0969
$f_{0}$	0.275	0.0898	0.122	0.0573	**0.0367**
*m*	0.342	0.0992	0.167	0.0870	**0.0354**
$d_{off}$	0.156	0.0821	0.0834	0.0777	**0.00256**
$p_{1}$	0.279	0.115	0.173	0.0971	**0.0314**
$p_{2}$	0.267	0.0885	0.121	0.0600	**0.0373**

**Table A3:** MAXAE on test set. Smaller is better.

	ResNet	BNN	MDN	PBNN	BayesFlow
$D_{L}$	1.159	0.293	0.792	0.459	**0.261**
$f_{0}$	0.984	0.251	0.495	0.307	**0.0993**
*m*	1.304	0.359	0.634	0.477	**0.123**
$d_{off}$	0.361	0.243	0.201	0.276	**0.0215**
$p_{1}$	1.147	0.383	0.692	0.458	**0.104**
$p_{2}$	0.968	0.255	0.491	0.320	**0.101**
